# Patient and therapist experiences of exposure therapy for anxiety-related disorders in pregnancy: qualitative analysis of a feasibility trial of intensive versus weekly CBT

**DOI:** 10.1192/bjo.2023.585

**Published:** 2023-10-12

**Authors:** Fiona L. Challacombe, Katherine Sabin, Samantha Jacobson, Rose Tinch-Taylor, Laura Potts, Ben Carter, Vanessa Lawrence

**Affiliations:** Section of Women's Mental Health, Health Service and Population Research Department, Institute of Psychiatry, Psychology & Neuroscience, King's College London, London, UK; Department of Biostatistics and Health Informatics, Institute of Psychiatry, Psychology & Neuroscience, King's College London, London, UK; and King's Clinical Trials Unit, Institute of Psychiatry, Psychology & Neuroscience, King's College London, London, UK; Health Service and Population Research Department, Institute of Psychiatry, Psychology & Neuroscience, King's College London, London, UK

**Keywords:** Anxiety disorders, cognitive–behavioural therapy, pregnancy, exposure therapy, obsessive–compulsive disorder

## Abstract

**Background:**

Approximately 15% of pregnant women experience anxiety disorders. Effective treatments exist but their acceptability during pregnancy, particularly exposure therapy, is not known.

**Aims:**

To understand patient and therapist experiences of time-intensive and weekly exposure-based therapy for anxiety disorders delivered during pregnancy. Trial registration: ISRCTN81203286.

**Method:**

In-depth interviews were conducted with patients and therapists who had taken part in a feasibility trial of predominantly online time-intensive versus weekly cognitive–behavioural therapy in pregnancy in a primary care setting in the UK. Data were analysed using reflexive thematic analysis.

**Results:**

In total, 45 women participating in the trial and 6 therapists who had delivered the treatments were interviewed. Five themes were developed from the data that showed convergence from therapist and patient perspectives: ‘Acquiring tools to navigate the perinatal period’; ‘Motivated yet constrained by pregnancy’; ‘Having the confidence to face fears and tolerate uncertainty’; ‘Momentum with the need for flexibility’; ‘Being removed from the face-to-face world’.

**Conclusions:**

Exposure therapy is acceptable and helpful in pregnancy and can lead to lasting gains. Exposure is a key element of treatment and needs to be confidently conducted by therapists with perinatal knowledge and expertise. Treatments need to consider the unfolding context of pregnancy. The momentum of intensive therapy can lead to rapid improvements, but is demanding for both patients and therapists, especially fitting round other commitments. Online treatments can work well and are a good fit for perinatal women, but this needs to be balanced with the need for social connection, suggesting a hybrid model is the ideal.

Pregnancy is a time of dynamic physical, emotional and social change. Mental health difficulties are prevalent in pregnancy, with anxiety-related problems being particularly common, affecting approximately 15% of women.^1^ Anxiety-related difficulties include a range of presentations, encompassing obsessive–compulsive disorder (OCD), post-traumatic stress disorder (PTSD), social anxiety and panic disorder. These problems are often accompanied by pregnancy-related anxiety.^2^ The disorders may exist prior to conception, but for many women the experience of clinical anxiety is new in pregnancy.^3^ Women with prior mental health difficulties, additional social stressors and a history of loss may be more likely to experience poor mental health in pregnancy, and this can be compounded by stressful events that occur within the pregnancy, including pregnancy complications.^4,5^ Persistent anxiety is not only distressing and disabling for women, but can potentially affect the pregnancy, birth and developing child.^6^ Large cohort studies have highlighted subsequent effects of antenatal anxiety on offspring during childhood^7^ which may be related to both genetic and environmental factors, including the intrauterine environment.^8^ A number of studies have also found antenatal anxiety to be associated with more challenging experiences of postnatal parenting and parental stress.^9,10^ Therefore, the need to treat anxiety effectively in pregnancy to alleviate the impact on women and offspring is of clear importance.

Women have a strong preference for psychological therapies in pregnancy, possibly owing to concerns about teratogenic effects of medications.^11^ Cognitive–behavioural approaches for anxiety-related problems have a substantial evidence base,^12^ but their use in pregnancy is much less researched. Studies have emphasised the need to adapt treatments to the perinatal context, with women valuing therapist knowledge and flexibility in delivery of sessions.^13^ However, high drop-out rates are often observed in perinatal treatment studies,^14^ which may be due to a range of factors, including financial and logistical barriers, the increasing physical impacts of pregnancy, non-tailored care, stigma and lack of trust in professionals. Pregnant women often have many other competing demands to manage, such as medical appointments, other caregiving responsibilities and paid employment.^15^

Time-intensive treatments deliver the treatment protocol over a shorter period (typically 1–2 weeks) in longer and more frequent sessions, and have been found to be effective in the treatment of OCD, PTSD, social anxiety and panic disorder.^16^ This form of delivery has been found to be effective and acceptable for women with postpartum OCD^17^ and may be a good fit given the time frame of pregnancy. An effective treatment dose earlier on could alleviate distress in the remainder of the pregnancy and thereby potentially improve a range of outcomes. Therapy delivered in fewer, longer sessions may be easier to manage around other demands and so lead to better adherence and engagement. One key component of therapy for anxiety problems is *in vivo* exposure to feared stimuli (for example to traumatic memories, unwanted sensations, feared situations) to facilitate new learning, which is an important element of treatment efficacy.^18^ However, therapist reluctance to conduct exposure has been documented in non-perinatal studies,^19^ and in surveys pregnancy is cited as a common reason for withholding or delaying treatment.^20,21^ In one study, almost half of surveyed therapists reported having heard that treatment of PTSD in pregnancy was harmful and over 30% reported reluctance to treat pregnant women.^21^ Therapist beliefs about upsetting or unbalancing the patient or harming the fetus can be activated, leading to therapist avoidance of this exposure. Concern is founded on the notion that exposure treatments may elevate anxiety and this may confer a risk to the developing fetus, given the known burden of chronic prenatal stress and anxiety.^7^ However, this approach disregards the fact that women are already experiencing high levels of anxiety at the start of therapy and the evidence that anxiety reduces with exposure interventions.^22^ The common exclusion of pregnant women from cognitive–behavioural therapy (CBT) trials has compounded this issue. Given the high prevalence of anxiety-related problems in pregnancy and the existence of effective treatments, it is important to explore the perspectives of women and therapists. This study aimed to understand the views of women and treating therapists on the experiences of providing and receiving cognitive–behavioural treatment including exposure in pregnancy. It also aimed to understand whether time-intensive treatment was a helpful and acceptable adaptation.

## Method

### Study design

Participants were women and therapists who had been part of a feasibility randomised controlled trial testing two forms of delivery of CBT for anxiety problems in pregnancy, delivered in a primary care setting.^23^ Women with a primary diagnosis of an anxiety disorder (OCD, PTSD, social anxiety or panic disorder) received predominantly online time-intensive or standard weekly CBT. The majority of treatment took place in pregnancy, with a follow-up at 1 month postpartum. Time-intensive CBT involved undertaking an initial 8–10 h of therapy in the first 2 weeks of treatment, with subsequent antenatal sessions at monthly intervals, whereas weekly CBT comprised 1 h per week throughout pregnancy. Thus, 8–11 h of antenatal contact time was planned in both arms, with an additional 1 h postpartum follow-up. A limited number of additional CBT sessions were allowed if women required further contact postpartum. All women who had taken part were invited to complete a final research assessment at approximately 3 months postpartum and invited to participate in a semi-structured interview as part of this.

**Fig. 1:**
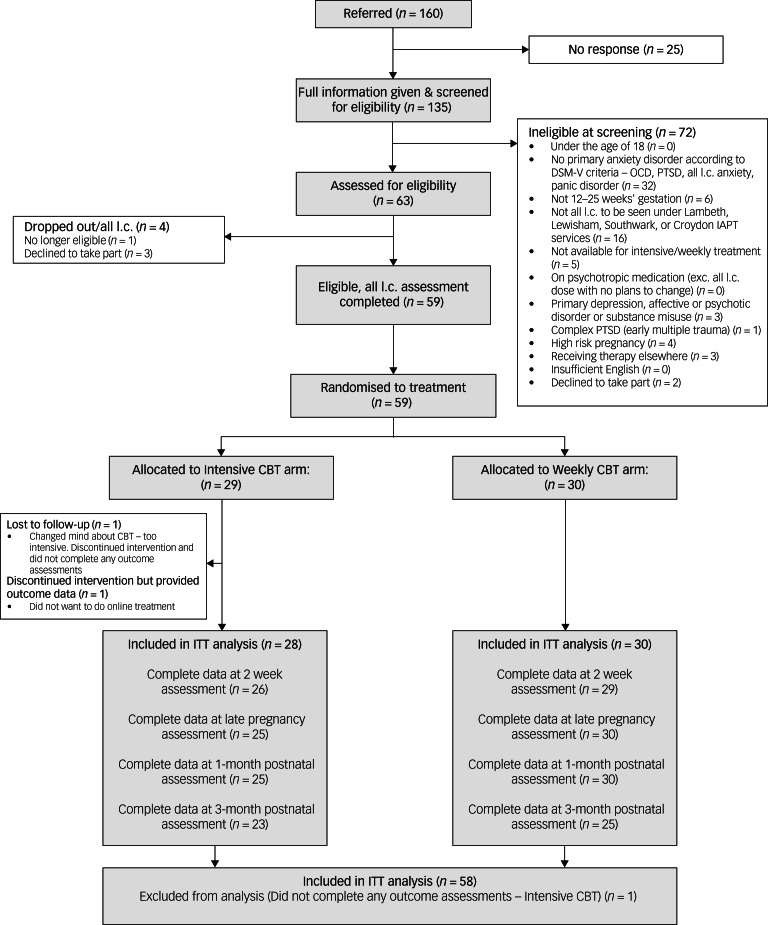
Flow of participants through the study. IAPT, Improving Access to Psychological Therapies; exc, excluding; PTSD, post-traumatic stress disorder; CBT, cognitive–behavioural therapy; ITT, intention to treat.

Women gave written informed consent to take part in the research and interview at the outset of the trial. Recruitment took place between 1 September 2019 and 24 September 2021 (Fig. 1). Six out of eight possible therapists were approached to take part – one of the eight had left the service and one was conducting this qualitative analysis (F.L.C.). All therapists were experienced in working with perinatal women prior to the trial, with between 3 and 25 years of post-qualification clinical experience of CBT and all were female.

Our qualitative approach was underpinned by an interpretivist ideology that aimed to understand experiences of therapy from the perspectives of those who will provide and receive it. Participant interviews were conducted by two female Masters level graduate students not otherwise involved in the trial (K.S. and S.J.) who had undertaken qualitative methods training as part of their Masters degrees. At the outset of participant interviews, the interviewer was masked to group participation (but was unmasked as the participants discussed their experiences, including the intensive or weekly format). Interviews took place online over Microsoft Teams and were recorded for transcription. Interviews were not returned to participants. Interviewees were aware that the interviewers were undertaking the project as part of the process evaluation of the ADEPT trial^24^ and were aware of the background of the researchers. Data were analysed by K.S. and F.L.C., who is an active clinician and researcher in the field of perinatal anxiety and chief investigator of the trial. Regular discussion on data collection and analysis took place with V.L., who is an expert in qualitative methods and member of the research team. The consolidated criteria for reporting qualitative research (COREQ) guideline was used to support the transparent reporting of the research.^25^

### Ethical considerations

A multicentre, parallel-group feasibility randomised controlled trial of standard (weekly) versus intensive exposure-based CBT (1:1) was conducted. A full protocol was published consistent with the SPIRIT guidance.^23^ The authors assert that all procedures contributing to this work comply with the ethical standards of the relevant national and institutional committees on human experimentation and with the Helsinki Declaration of 1975, as revised in 2008. All procedures involving human subjects/patients were approved by London-Surrey Borders research Ethics Committee (19/LO/0622).

### Interviews

The topic guides for the mother interviews were developed from the literature, discussions with experts by experience and clinicians working in this field. Possible additional prompts and observations were noted by F.L.C. and K.S. as the trial continued. Owing to the timing of the trial, questions were added about online therapy and the impact of the COVID-19 pandemic. The aim of the mother interviews was to explore the experiences of women and to identify any benefits and/or harms from the intervention or participation in the study (see the Supplementary material available at https://doi.org/10.1192/bjo.2023.585 for topic guides). The topic guide for the therapist interviews was developed with the research team. Data collection and analysis overlapped, allowing us to review when we had achieved information power^26^ and collected sufficiently rich and diverse data to answer our research question.

### Data analysis

We conducted reflexive thematic analysis using inductive codes to develop conceptual themes, using NVivo version 12 for Windows qualitative analysis software (QSR International) to help organise the data. The six phases outlined by Braun & Clarke were followed.^27^ Transcripts were first checked against interview recordings for accuracy. Analysis began with re-reading of transcripts and listening to audio files for immersion. Two of the researchers (K.S. and F.L.C.) read the first three participant transcripts repeatedly to immerse themselves in the data; they then independently developed initial codes and provisional themes and discussed with a third researcher (V.L.) to examine different interpretations of the data and enhance reflexivity. Reflexive thematic analysis values researcher subjectivity while recognising the importance of critically reflecting on the knowledge that is produced. Involving others in the analysis helped the lead author to understand how her personal experiences of pregnancy, motherhood, therapy and perinatal mental health research, and role as principal investigator of the study, might have influenced expectations, assumptions and interpretations. This process was repeated after 10 interviews had been coded. Therapist data were coded by F.L.C. and discussed with V.L., informing provisional themes that overlapped with the patient data. The provisional themes were discussed in the research team and the perspectives of both groups of participants were amalgamated to develop rich and nuanced overarching themes. Remaining data were coded into themes and subthemes relating to participant and therapist experiences and attitudes towards treatment.

### Patient and public involvement

Women who had experienced perinatal mood and anxiety disorders were involved in the design of the study, and a lived experience group was established to advise on the progress and results of the study, including feedback on qualitative results. This group advised that the mother interview topic guide was suitable and at the results stage advised that that it was particularly important to take the practicalities of intensive therapy into account for women. A person with lived experience was also part of the data monitoring committee.

## Results

In total, 45 women who had participated in the ADEPT trial (of a total possible 59 randomised) were interviewed, including one woman who had dropped out of treatment. Two women did not have time for the interview but provided quantitative data for the research assessment; the remaining women were not available for follow-up. Six (of a possible eight) therapists who had conducted the vast majority of intervention in the trial were interviewed about their experiences. All therapists had delivered both weekly and time-intensive treatments (Table 1).

**Table 1:** Characteristics of participants

	Weekly CBT (*n* = 23)	Intensive CBT (*n* = 22)
**Patients (*n* = 45)**		
Anxiety disorder, *n*		
OCD	7	6
PTSD	9	8
Social anxiety disorder	2	3
Panic disorder	5	5
First-time parent, %	63.6	56.5
Ethnicity, *n*^a^		
White	19	17
Black	1	1
Asian	1	1
Mixed	1	3
Age, years: mean (s.d.)	33.1 (4.2)	31.1 (10.8)
**Therapists (all female) (*n* = 6)**		
Clinical psychologist	2	
Counselling psychologist	1	
CBT therapist	3	

CBT, cognitive–behavioural therapy; OCD, obsessive–compulsive disorder; PTSD, post-traumatic stress disorder.

a. One person declined to give their ethnicity.

### Qualitative themes and subthemes

Themes were generated using data from both women and therapists. Themes and subthemes are summarised in Table 2 and discussed in detail below. Participants’ names are replaced with pseudonyms, and disorder and mode of therapy (WCBT, weekly CBT; INT, intensive CBT) are shown.

**Table 2:** Themes and subthemes of the qualitative analysis

Theme	Subthemes
Acquiring tools to navigate the perinatal period	Better equipped for birth and parenthood
	Therapy as a means to improve relationships
Motivated yet constrained by pregnancy	Pregnancy context as integral to therapy
	Overcoming physical and social barriers
Having the confidence to face fears and tolerate uncertainty	Therapeutic relationship as the foundation for exposure
	Sitting with uncertainty
Momentum with the need for flexibility	Momentum of intensive CBT
	Being responsive to the individual situation
Being removed from the face-to-face world	Flexibility and barriers of online working
	Limitations to social connection

#### Theme 1: Acquiring tools to navigate the perinatal period

An overarching theme of using therapy as a means to navigate the perinatal period was described. Women spoke of the use of tools and techniques to help with their anxiety, with some mentioning specific examples such as reducing excessive internet-based reassurance-seeking or using stimulus discrimination in PTSD. The normalising of anxiety or intrusive thoughts, allowing women to understand their experiences, was mentioned by several as helpful. For those with panic disorder, understanding that sensations were not dangerous was important. Women across disorder groupings mentioned mapping out the problem (formulation) as helpful to understanding and changing their thoughts and behaviours. Women liked the individual and flexible approach to their difficulties:

‘I definitely did think it was a positive experience for me in terms of addressing my anxiety and she gave me some techniques when I'm experiencing particularly, like, physical symptoms to bring myself back into the moment. And that was really helpful. And she taught me some really good techniques to help me deal with my anxiety.’ (Mary: PTSD-WCBT)

Some women described limitations in terms of improvements in some aspects of mental health, highlighting that not all aspects were addressed by the therapy. Many also recognised that therapy was the beginning of an ongoing process and the need to keep actively applying the tools to continue to make progress. Therapists also reflected on the improvements seen in many of their patients.

##### Better equipped for birth and parenthood

Several women reported feeling more confident with the birth process and in asking for assistance if they needed, as well as feeling calmer in general. Women had spoken about reducing the impact of their anxiety on parenting as a motivation for seeking treatment and several mentioned benefits overall to their sense of anxiety and confidence as a parent, and that they had become more relaxed. Some described specific strategies learned in therapy that they were applying to parenting:

‘I do tend to worry about how good a mother I am. But it has helped me to have strategies, and, instead of worrying about it, to spend more time engaging with her.’ (Rona: Panic-INT)

##### Therapy as a means to improve relationships

Many women mentioned a positive effect of therapy on relationships with partners, either by being involved in the sessions and gaining more understanding of the problem themselves or when women started to apply what they had learned in treatment, for example by partners being less involved in reassurance-seeking or, indirectly, by the mother being calmer in general:

‘I think it had a positive effect on my closest relationships, because in pregnancy, especially with lockdown as well, they were the people that had to deal with my intrusive thoughts when I shared them, and my worries every day.’ (Ruby: OCD-INT)

Therapists reflected on the importance of considering partner involvement as the women progressed through therapy.

#### Theme 2: Motivated yet constrained by pregnancy

##### Pregnancy context as integral to therapy

Therapists and women reflected on ways that the time frame and context of pregnancy were interwoven with the therapy. Women felt pregnancy was a good time for them to have had the therapy to help them prepare for birth and parenthood:

‘to have someone with you as it happens and to say “I've got a scan next week. How do I prepare for this?”, you know, and to have someone giving you those kind of real-life live tools and tricks made all the difference, like, rather than having it before you get pregnant or whatever and then trying to apply it in a time where, you know, a lot's going on. So, for me it was really, really helpful having it at that point.’ (Jemima: PTSD-WCBT)

Some women who had had intensive CBT, particularly those with PTSD, commented that this was a particularly good time to work on things and make rapid progress, in order to enjoy the pregnancy more.

Women spoke about the importance of committing to the therapy, making the most of it and trying to prioritise it at this time. For some, this was recognised more retrospectively. Therapists noted the importance of starting therapeutic work as early as possible in the pregnancy, particularly in the case of trauma work. If additional physical problems arose as pregnancy progressed then this made delivery of treatment more difficult as women were potentially increasingly anxious and preoccupied with those, with less capacity to challenge their anxiety, and some had additional maternity appointments to negotiate. Normalisation of experiences was a very helpful tool for therapists and they recognised a need for ongoing reflection on what was normal or excessive in the context of pregnancy:

‘It's really important to understand how their pregnancy's affecting their anxiety and to really think about how their anxiety is affecting their pregnancy as well because, you know, in my experience to date and through the trial people's anxiety was much higher than it was outside of their pregnancy.’ (Therapist 2)

##### Overcoming physical and social barriers

Many women mentioned tiredness during pregnancy as affecting the sessions or homework to some extent, particularly as the pregnancy progressed. Having intensive sessions was particularly tiring for some, but others felt that these came at the right time before they became tired later in pregnancy. Some, but not all, of the women felt that being pregnant may have made them more emotionally labile, affecting both their anxiety and the therapy. Therapists frequently noted that their patients were very busy, and fitting therapy and homework around paid employment was difficult. This was mirrored by comments from women in both intensive and weekly therapy, with intensive therapy placing more demands on women regarding homework tasks. Some thought the intensive therapy was easier to fit in because it was short term, but some from this group and many in the weekly group felt that weekly sessions were more practical to fit around work demands:

‘I did struggle with the amount of homework I had to do. I think because I was working full-time as well, yeah, I struggled a little bit with that, so I'd say that was probably the one thing that I would like to have done differently.’ (Amal: PTSD-WCBT)

Furthermore, therapists noted that women experiencing additional issues may have found it harder to engage with the treatment:

‘You know people are struggling with symptoms and stuff like physical symptoms. I think that you need to be a bit more flexible and unfortunately services aren't always very flexible.’ (Therapist 2)

Therapists pointed out that stability of circumstances is a necessary condition to use therapy and noted their impression that those mothers who were also dealing with social problems such as insecure housing did less well. Therapists commented that this was often intersectional, with more women from minority ethnic backgrounds experiencing these problems. They emphasised the importance of flexibility in service provision and cancellation policies to allow women time to trust and engage, of particular importance for groups experiencing marginalisation.

#### Theme 3: Having the confidence to face fears and tolerate uncertainty

##### Therapeutic relationship as the foundation for exposure

Therapists endorsed the use of exposure techniques with pregnant patients and noted the importance of explaining the rationale clearly and being confident with patients in this part of the therapy. Pregnant women were described as being both more motivated and more cautious than non-perinatal patients, and they were mindful of any theoretical impact on the baby, which was sometimes discussed in treatment:

‘being really clear on what the work involves and being really confident in how some of the work involves exposure and increasing your anxiety and how that's safe is really important in this population…’ (Therapist 2)

Women themselves repeatedly emphasised the importance of the therapeutic relationship in helping them approach their fears during exposure exercises, and the subsequent value of having done this:

‘I feel braver and I feel like I can tolerate the discomfort of “Oh God, is this going to be okay?” more because of having done it with support…’ (Tania: Panic-WCBT)

‘I really hated that [exposure] with a fiery passion, but I did it and I think it was really useful…’ (Sarah: PTSD-WCBT)

Therapists modified exposure according to what the patients could physically and emotionally manage and noted a view that conducting exposure exercises very late in pregnancy (i.e. after 35 weeks) was not advisable, owing to the uncertainty surrounding how many sessions remained before birth and the need to focus and prepare for this event.

##### Sitting with uncertainty

All therapists noted that the work of supporting exposure sometimes brought up internal therapist beliefs and doubts about harm to the baby. They did not feel that this changed what they did in treatment but it was something they had to tolerate in the work and this was important given that is also what is being asked of the patients. Sometimes the fears were ‘contagious’ to therapists, who then questioned their judgements, particularly where pregnancy complications arose such as gestational diabetes:

‘Sometimes, you know, we can never guarantee you a pregnancy goes to full term, we can never guarantee there won't be complications and I think sitting with that can be really unhelpful, hard, sorry, can be really, yeah, difficult, I think, for us…’ (Therapist 4)

#### Theme 4: Momentum with the need for flexibility

##### Momentum of intensive CBT

There was clear feedback from the perspectives of both women and therapists that intensive CBT engendered a fast pace of change and high momentum. Several women spoke of the benefits of intensive sessions, with things moving quickly and efficiently forward without the need for long recaps. Longer sessions allowed for coverage of a lot of material that was picked up quickly in the following session. They spoke of making quick progress owing to the amount of therapist contact. Some noted that their motivation and commitment were higher as a result of having frequent sessions. Feeling better in a shorter amount of time was also very reinforcing, and some women felt that this had allowed them to enjoy their pregnancy more by having completed the treatment earlier. By contrast some women in the weekly arm took longer to get going and notice improvements, for example noting that one missed session meant a long time before seeing the therapist again:

‘it's definitely at the forefront of your mind and to make the most of the time that you've got with the therapist. I think the intensity lends itself to really committing to using the strategies, I would say.’ (Ruby: OCD-INT)

Therapists noted a quicker response in intensive CBT and several reported a strong therapeutic alliance due to the large amount of time spent together in the first weeks. Treatment was particularly effective if women had been able to prioritise the therapy and had blocked time out for it. But women and therapists also noted that for some, more time was needed for homework tasks such as reading, carrying out experiments and emotional processing of the material between sessions. In addition to noting the challenges for patients, therapists noted the challenges of fitting sessions in their diaries and that services would need to have flexibility to offer this as a standard treatment option:

‘I think challenges logistically fitting it in, like, that was the – you know, both from my diary and their diary trying to just actually fit in sessions [was] quite tricky.’ (Therapist 3)

Therapists reflected that for women who were more difficult to engage or were dealing with other issues, having a burst of sessions and an initial ‘therapeutic dose’ may have been very useful:

‘She was able to enjoy the rest of her pregnancy a lot more, so in that way it was really beneficial. Whereas if we hadn't done that, it would have taken, yeah, 10 weeks. And that, yeah, that would have meant more time feeling distressed.’ (Therapist 1)

##### Being responsive to the individual situation

Therapists noted that in the intensive arm they had to be focused and were less able to respond to concerns as they arose, whereas women who received weekly treatment often reported this as a benefit of this approach:

‘It was actually helpful, so it's like obviously I was, I had a bit of a difficult pregnancy, so I was like worrying, every week there were obstacles coming up, so I knew I could talk about it in my session, that I had my session coming up.’ (Claire: PTSD-W)

Although most patients maintained the gains made during pregnancy in both treatment arms and did not need further postnatal sessions, some did require them, either because of further incidents that occurred, such as a traumatic birth, or if the original problem was related to perinatal loss or bereavement. Therapists highlighted the need for clinical flexibility in these circumstances, which are not uncommon in the perinatal period.

#### Theme 5: Being removed from the face-to-face world

##### Flexibility and barriers of online working

Women had mixed feedback regarding online therapy – many felt that it was more practical around work and the physical demands of attending (any sort of) therapy. However, for trust and engagement it was helpful to meet the therapist at least once in the treatment where that had been possible. Some but not all felt that the therapeutic alliance was stronger having met face to face:

‘we got on well together and things, but I do think it's probably just one step harder doing it through a screen. Having said that, I think any negatives are outweighed by the benefit of not having to go anywhere, like just being able to do a two-hour session rather than adding in travel time.’ (Fionn: PTSD-INT)

This was directly mirrored by the experience of therapists, who felt that the online sessions allowed some to access the service who would not otherwise have engaged:

‘And I think if I'd been seeing some of those women in person, they just wouldn't have come, like they would have cancelled, they just wouldn't have come to the session. But actually seeing them remotely meant that they could come even if it was really difficult to get out that day.’ (Therapist 2)

Similarly, online exposure exercises worked well for some women, but others felt that this aspect was more compromised and that more active work had or would have taken place in face-to-face meetings. This may have been mediated by the impact on the therapeutic alliance, or that more spontaneity occurred in face-to-face sessions.

### Limitations to social connection

The online delivery of the therapy was driven by the impact of the COVID-19 pandemic. Women spoke of missing out on social connection and family support during the pandemic, which made their anxiety and their pregnancies more difficult. Therapists and women noted that being unable to connect directly with others meant that normalising information was not available and that loneliness and isolation in the postnatal period was an additional problem they had to face.

Several therapists noted that for some women, particularly those with OCD, the pandemic made the problem more difficult to treat as it reinforced avoidance and made conducting exposure exercises very difficult:

‘With OCD, it was a bit tougher to get them to maybe do exposure and because they felt validated with the with the COVID concerns…’ (Therapist 1)

## Discussion

This study is the first to explore in detail the parallel experiences of women and therapists of undertaking exposure-based CBT for anxiety problems during pregnancy. We found that women were positive about undertaking CBT during pregnancy and were motivated, often wanting to improve their anxiety and develop tools to reduce any potential impact on their baby. Generally, women reported benefits from having treatment on their mental health and on their functioning more broadly, as well as on relationships.

Previous research has identified the importance of tailored treatments in the perinatal period and of the treating therapists having specialist knowledge.^28^ Our findings support this and demonstrate the particular importance of perinatal expertise in delivering exposure-based treatments confidently with pregnant women. Knowledge and understanding of what is normal in the perinatal period is crucial for therapists to be able to contextualise their patients’ experience, as well as knowledge of the impact of pregnancy complications and the pregnancy history on patients’ needs. Expertise and skills in anxiety treatments is also of obvious importance, with the ability to model a tolerance of uncertainty being key for therapists to deliver effective treatment.^19^

Our findings indicate that the context of pregnancy was of also of importance to how treatments were conducted. Cognitive–behavioural approaches are very active and the logistical challenges of finding time and emotional capacity to undertake treatment is an important consideration. Good attendance and completion of homework are related to better outcomes in CBT for anxiety problems.^29^ The frequent sessions of intensive CBT can put pressure on time and energy for homework, and additional social stressors are likely to have a further impact on resources to complete between-session tasks:^15^ both of these factors were highlighted by therapists in this study. Therapist expectations of homework for perinatal women may therefore need to be modified, given the large demands on women's time and capacity.^30^ Pregnancy can be eventful and ideally the therapeutic context will be responsive to this.^30^ The need for flexibility in service provision and cancellation policies was an important finding from our results.

This analysis indicated that intensively delivered therapy can be demanding for women and therapists, but our study also highlighted that for most women the momentum of this approach meant that they experienced a quick benefit, which was important in the limited time frame of pregnancy. Therefore, this seemed to be an acceptable approach to treatment delivery. It is possible that having a briefer treatment that is effective early in pregnancy could be particularly useful for those who are managing significant external pressures or subsequent pregnancy complications that may make adherence to a longer programme more difficult.

Although online treatments for mental health problems can be effective, there may be unique considerations for perinatal women.^31^ Our findings indicated clear pros and cons to online delivery of therapy, with women and therapists offsetting accessibility against engagement. Women highlighted the benefit of having at least some face-to-face contact, perhaps suggesting an ‘ideal’ model of hybrid work. This seems important given the key role of loneliness in maintaining perinatal mental health difficulties.^32^

### Strengths and limitations

A strength of this study was that we were able to include the perspectives of a relatively large number of women and their therapists. However, not all women who participated in the trial gave feedback on their experiences, which may have led to less representation of more negative experiences. Other limitations were that themes were not checked with participants and the sample overall lacked ethnic diversity. The therapy took place during the COVID-19 pandemic, which affected women's mental health and necessitated online or hybrid therapy and is important context for this particular study. However, similar indications of acceptability and efficacy were found in a pre-pandemic face-to-face trial of intensive therapy for postpartum women with OCD.^17^ Furthermore, many talking therapies services are continuing to provide online and hybrid delivery, which may be particularly popular with perinatal women, and so results may align well with current (post-pandemic) clinical practice.

### Further research and service implications

Given that the exposure-based treatments were generally considered acceptable and useful by the pregnant women in our study, the effectiveness of these therapies should continue to be rigorously tested in definitive trials across a number of settings. Therapist perinatal expertise and service flexibility must be ensured to enable the successful delivery of CBT in pregnancy.

## Supporting information

Challacombe et al. supplementary material Challacombe et al. supplementary material

## Data Availability

The data that support this study are available on request from the corresponding author (F.L.C.). The data are not publicly available as they contain information that could compromise the privacy of research participants.

## References

[1] Dennis C-L, Falah-Hassani K, Shiri R. Prevalence of antenatal and postnatal anxiety: systematic review and meta-analysis. Br J Psychiatry 2017; 210: 315–23. 28302701 10.1192/bjp.bp.116.187179

[2] Nath S, Lewis L, Bick D, Demilew J, Howard L. Mental health problems and fear of childbirth: a cohort study of women in an inner-city maternity service. Birth 2021; 48: 230–41. 33733519 10.1111/birt.12532

[3] Fairbrother N, Janssen P, Antony MM, Tucker E, Young AH. Perinatal anxiety disorder prevalence and incidence. J Affect Disord 2016; 200: 148–55. 27131505 10.1016/j.jad.2015.12.082

[4] Charrois EM, Mughal MK, Arshad M, Wajid A, Bright KS, Giallo R, et al. Patterns and predictors of depressive and anxiety symptoms in mothers affected by previous prenatal loss in the ALSPAC birth cohort. J Affect Disord 2022; 307: 244–53. 35339570 10.1016/j.jad.2022.03.055

[5] Biaggi A, Conroy S, Pawlby S, Pariante CM. Identifying the women at risk of antenatal anxiety and depression: a systematic review. J Affect Disord 2016; 191: 62–77. 26650969 10.1016/j.jad.2015.11.014 PMC4879174

[6] Ding X-X, Wu Y-L, Xu S-J, Zhu R-P, Jia X-M, Zhang S-F, et al. Maternal anxiety during pregnancy and adverse birth outcomes: a systematic review and meta-analysis of prospective cohort studies. J Affect Disord 2014; 159: 103–10. 24679397 10.1016/j.jad.2014.02.027

[7] O'Donnell KJ, Glover V, Barker ED, O'Connor TG. The persisting effect of maternal mood in pregnancy on childhood psychopathology. Dev Psychopathol 2014; 26: 393–403. 24621564 10.1017/S0954579414000029

[8] Ahmadzadeh YI, Schoeler T, Han M, Pingault J-B, Creswell C, McAdams TA. Systematic review and meta-analysis of genetically informed research: associations between parent anxiety and offspring internalizing problems. J Am Acad Child Adolesc Psychiatry 2021; 60: 823–40. 33675965 10.1016/j.jaac.2020.12.037 PMC8259118

[9] Thiel F, Iffland L, Drozd F, Haga SM, Martini J, Weidner K, et al. Specific relations of dimensional anxiety and manifest anxiety disorders during pregnancy with difficult early infant temperament: a longitudinal cohort study. Arch Womens Ment Health 2020; 23: 535–46. 31927695 10.1007/s00737-019-01015-w PMC7369131

[10] Huizink AC, Menting B, Moor MHM, Verhage ML, Kunseler FC, Schuengel C, et al. From prenatal anxiety to parenting stress: a longitudinal study. Arch Womens Ment Health 2017; 20: 663–72. 28634716 10.1007/s00737-017-0746-5 PMC5599437

[11] Arch JJ. Cognitive behavioral therapy and pharmacotherapy for anxiety: treatment preferences and credibility among pregnant and non-pregnant women. Behav Res Ther 2014; 52: 53–60. 24326075 10.1016/j.brat.2013.11.003

[12] Norton PJ, Price EC. A meta-analytic review of adult cognitive-behavioral treatment outcome across the anxiety disorders. J Nerv Ment Dis 2007; 195: 521–31. 17568301 10.1097/01.nmd.0000253843.70149.9a

[13] O'Mahen H, Fedock G, Henshaw E, Himle JA, Forman J, Flynn HA. Modifying CBT for perinatal depression: what do women want? A qualitative study. Cogn Behav Pract 2012; 19: 359–71.

[14] Orhon FS, Soykan A, Ulukol B. Patient compliance to psychiatric interventions and course of postpartum mood disorders. Int J Psychiatry Med 2007; 37: 445–57. 18441631 10.2190/PM.37.4.g

[15] Smith MS, Lawrence V, Sadler E, Easter A. Barriers to accessing mental health services for women with perinatal mental illness: systematic review and meta-synthesis of qualitative studies in the UK. BMJ Open 2019; 9(1): e024803. 10.1136/bmjopen-2018-024803 PMC6347898 30679296

[16] Remmerswaal KCP, Lans L, Seldenrijk A, Hoogendoorn AW, van Balkom AJLM, Batelaan NM. Effectiveness and feasibility of intensive versus regular cognitive behaviour therapy in patients with anxiety and obsessive-compulsive disorders: a meta-analysis. J Affect Disord Rep 2021; 6: 100267.

[17] Challacombe FL, Salkovskis PM, Woolgar M, Wilkinson EL, Read J, Acheson R. A pilot randomized controlled trial of time-intensive cognitive-behaviour therapy for postpartum obsessive-compulsive disorder: effects on maternal symptoms, mother-infant interactions and attachment. Psychol Med 2017; 47: 1478–88. 28137316 10.1017/S0033291716003573

[18] Maguire PN, Clark GI, Wootton BM. The efficacy of cognitive behavior therapy for the treatment of perinatal anxiety symptoms: a preliminary meta-analysis. J Anxiety Disord 2018; 60: 26–34. 30388545 10.1016/j.janxdis.2018.10.002

[19] Meyer JM, Kelly PJ, Deacon BJ. Therapist beliefs about exposure therapy implementation. Cogn Behav Ther 2020; 13: e10.

[20] Meyer JM, Farrell NR, Kemp JJ, Blakey SM, Deacon BJ. Why do clinicians exclude anxious clients from exposure therapy? Behav Res Ther 2014; 54: 49–53. 24530499 10.1016/j.brat.2014.01.004

[21] Hendrix YMGA, Sier MAT, Baas MAM, van Pampus MG. Therapist perceptions of treating posttraumatic stress disorder in pregnancy: the VIP study. J Trauma Stress 2022; 35: 1420–31. 35535472 10.1002/jts.22842

[22] Arch JJ, Dimidjian S, Chessick C. Are exposure-based cognitive behavioral therapies safe during pregnancy? Arch Womens Ment Health 2012; 15: 445–57. 22983422 10.1007/s00737-012-0308-9

[23] Challacombe FL, Potts L, Carter B, Lawrence V, Husbands A, Howard LM. Optimising psychological treatment for Anxiety DisordErs in Pregnancy (ADEPT): study protocol for a feasibility trial of time-intensive CBT versus weekly CBT. Pilot Feasibil Stud 2021; 7(1): 101. 10.1186/s40814-021-00838-8 PMC8085465 33931111

[24] Challacombe F, Tinch-Taylor R, Sabin K, Potts L, Lawrence VL, Howard LM, et al. Exposure-based cognitive-behaviour therapy for anxiety disorders in pregnancy (ADEPT): results of a feasibility randomised controlled trial and process evaluation. PsyArXiv [Preprint] 2023. Available from: https://psyarxiv.com/g42s5/. 10.1016/j.jad.2023.10.070 37848088

[25] Tong A, Sainsbury P, Craig J. Consolidated criteria for reporting qualitative research (COREQ): a 32-item checklist for interviews and focus groups. Int J Qual Health Care 2007; 19: 349–57. 17872937 10.1093/intqhc/mzm042

[26] Malterud K, Siersma VD, Guassora AD. Sample size in qualitative interview studies: guided by information power. Qual Health Res 2016; 26: 1753–60. 26613970 10.1177/1049732315617444

[27] Braun V, Clarke V. Successful Qualitative Research. SAGE Publications, 2013.

[28] Lever Taylor B, Kandiah A, Johnson S, Howard LM, Morant N. A qualitative investigation of models of community mental health care for women with perinatal mental health problems. J Ment Health 2021; 30: 594–600. 32000552 10.1080/09638237.2020.1714006

[29] Glenn D, Golinelli D, Rose RD, Roy-Byrne P, Stein MB, Sullivan G, et al. Who gets the most out of cognitive behavioral therapy for anxiety disorders? The role of treatment dose and patient engagement. J Consult Clin Psychol 2013; 81: 639–49. 23750465 10.1037/a0033403 PMC3990403

[30] Millett L, Taylor BL, Howard LM, Bick D, Stanley N, Johnson S. Experiences of improving access to psychological therapy services for perinatal mental health difficulties: a qualitative study of women's and therapists’ views. Behav Cogn Psychother 2018; 46: 421–36. 29081328 10.1017/S1352465817000650

[31] Komariah M, Amirah S, Faisal EG, Prayogo SA, Maulana S, Platini H, et al. Efficacy of internet-based cognitive behavioral therapy for depression and anxiety among global population during the COVID-19 pandemic: a systematic review and meta-analysis of a randomized controlled trial study. Healthcare 2022; 10(7): 1224. 35885751 10.3390/healthcare10071224 PMC9315502

[32] Adlington K, Vasquez C, Pearce E, Wilson CA, Nowland R, Taylor BL, et al. ‘Just snap out of it’ – the experience of loneliness in women with perinatal depression: a meta-synthesis of qualitative studies. BMC Psychiatry 2023; 23(1): 110. 36849948 10.1186/s12888-023-04532-2 PMC9970854

