# Supplementary material for: Patient and therapist experiences of exposure therapy for anxiety-related disorders in pregnancy: qualitative analysis of a feasibility trial of intensive versus weekly CBT
Source: BJPsych Open. 2023 Oct 12;9(6):e189. doi: 10.1192/bjo.2023.585 (PMC10594159; doi:10.1192/bjo.2023.585)
Supplement: Challacombe et al. supplementary material [file S2056472423005859sup001.docx]

**ADEPT STUDY INDICATIVE TOPIC GUIDE**

**MOTHER INTERVIEW CONDUCTED AT 3M POSTNATAL (OUTCOME POINT)**

OVERVIEW OF THE TREATMENT

- How has life changed since I saw you last? [or if first meeting How has life been in the last few months?]?
- Looking back can you remember what your expectations of the therapy were?”
- How did you find the treatment?
- What did you like or find helpful?
- What did you not like or find unhelpful?
- Was there anything you wanted more/less of?

What did you make of the exposure aspect?

- Was there anything you would do differently?
- Did the treatment meet your expectations?
- Do you think you got the most out of the treatment?
- If no, what got in the way?
- Was there anything about being pregnant which affected the treatment? (possible prompts: physical access to the service, physical symptoms, other appointments, content of the treatment, concentration, physical effects of the treatment etc).

FORMAT SPECIFIC QUESTIONS

- How did you feel about the format in which the therapy was delivered?
- How well did that work for you at that time?
- What was your experience of the intensive/weekly format (the one you did)?
- What do you think were the pros and cons of doing treatment that way?
- How did you feel about the length of the sessions?
- How did you feel about the frequency of the sessions?
- What about the number of sessions?
- Did you have a preference for this format?
- What do you think the other format would have been like (possible prompts: pace of change; fitting it in with other commitments; advantages/disadvantages?
- Any advice you would give pregnant women starting treatment?

OVERALL VIEWS OF TREATMENT IMPACT

- We are interested in what you think about the treatment and there are no right or wrong answers. Do you think that the treatment did affect your anxiety? In what way?
  - Did the treatment affect the birth/labour? In what way?
  - Did the treatment affect parenting? In what way?
  - Did the treatment have an effect on anything else (positive or negative)?
  - Did anything surprise you about the treatment?

VIEWS OF THE IMPACT OF COVID

- Do you think that the pandemic has affected your anxiety? Positive or negative? (possible prompts: being able to stick to a routine, excused from social situations, concerns about health of self or baby, not having family/friends support)
- Do you think COVID-19 had an effect on your experience of therapy?
- Did COVID-19 affect your experience of pregnancy? (midwifery appointments)
- Did COVID-19 affect the birth/labour experience? In what way? (partner support, atmosphere on the ward)
- Has COVID-19 affected parenting? In what way? (Childcare, routine)

END OF INTERVIEW FEASIBILITY SPECIFIC QUESTIONS

- What did you think about the way you were approached to take part in the study? (prompt – the way you found out about and were enrolled in the study)?
- What was your view of being randomised in the study, so that you received either weekly therapy or therapy over 10 weeks?
- Did you feel that the questionnaires and other measures were looking at the right things?
- Was there anything unhelpful about the questionnaires? Was it a reasonable number/time?
- Do you have any other feedback for the researchers about how they are running the study?

**THERAPIST INTERVIEW TOPIC GUIDE**

## Background Information:

- How long have you been working as a therapist?
- What kind of experience do you have working with CBT?
- Have you worked using intensive CBT prior to the trial?
- What kinds of psychological problems have you treated?

## Working with pregnancy

- What are the key differences in therapeutic work with pregnant women with anxiety disorders?
  - What modifications did you make?
- What were your experiences treating specific disorders in pregnancy?

## Doing exposure in pregnancy

- How was using exposure in treatments?
  - How did people respond?
  - What were the advantages of doing exposure therapy in pregnancy?
  - What were the disadvantages?
  - Did you make any modifications due to pregnancy?
- Do you think there is an ‘ ideal’ time to do exposure? What were your experiences/ what did you notice for the patients of doing it in different trimesters?
- What are the nuances of exposure in terms of the different disorders?
- Was there anything about delivering exposure in pregnancy that brought up any therapist beliefs for you?
- Did that have any impact on how you did CBT in pregnancy?

## Working intensively

- In general, how would you describe the differences between Intensive CBT and weekly CBT?
- What are some of the main challenges in administering intensive therapy?
  - What are some of the issues that have arisen?
  - Are there any disadvantages
  - Are there any particular difficulties due to the shorter time frame? (prompt if needed – is the focus different? How?
- What are the benefits as a therapist of intensive therapy?
- In terms of therapeutic outcomes, what are the main benefits of giving intensive CBT? Do the outcomes differ compared to standard CBT?
  - What are the components of this form of therapy that work best?
- Are there any particular demands on you as a therapist?
- How does the therapeutic alliance with a patient compare when it’s longer sessions over a shorter period? Are there any particular impacts on the therapeutic allicance?
- How does the motivation of the patient (in terms of commitment, perseverance and determination to stay in treatment) compare in intensive therapy to standard CBT?
- Trial focused on what works mostly in pregnancy – what did you notice about the effects postnatally?
  - Was the balance right? Should we focus all the sessions during pregnancy or continue afterwards
  - Whats the ideal model of therapy considering there’s only a certain amount of sessions available
  - What is the ideal amount of sessions you believe should be offered
- In this group, what additional challenges were the women facing (eg low SES, domestic abuse, housing problems?
  - What impact did it have on therapy?
    - Is the impact different in intensive vs weekly sessions
  - Therapy services are not always equally accessed, in your opinion what can we do to mitigate that?

## Working in the pandemic

- What was your experience like working/administering therapy during the pandemic?
  - How were you able to adapt intensive CBT during this time?
    - were your visits in person or virtual?
      - If virtual how does this affect the process of CBT?
  - What were the main challenges of administering therapy during the pandemic time? What were some of the benefits?
  - Did the outcomes of therapeutic effectiveness change?
    - Are patients more/less likely to do well and engage in therapy if it’s online?
- What are the challenges setting up appointments with pregnant women during the pandemic?

- In your opinion, how has COVID-19 impacted the anxiety levels in the patients you’ve seen?
- How has it impacted your experience as a therapist in terms of:
  - Working/collaborating with colleagues
  - Engaging with your patients and adapting to them
  - Getting emotional support for yourselves as therapists
- How does therapy differ when patients are doing stuff in their homes eg PTSD
- Were there particular challenges related to particular disorders e.g. social anxiety treatment during the pandemic

Any comments about working under the ADEPT trial as a therapist?

Any other comments about working as a therapist in the pandemic?
